# Evaluation of pulmonary hypertension and clinical status in dogs with heartworm by Right Pulmonary Artery Distensibility Index and other echocardiographic parameters

**DOI:** 10.1186/s13071-017-2047-2

**Published:** 2017-02-28

**Authors:** B. Serrano-Parreño, E. Carretón, A. Caro-Vadillo, Y. Falcón-Cordón, S. Falcón-Cordón, J. A. Montoya-Alonso

**Affiliations:** 1 Internal Medicine, Faculty of Veterinary Medicine, Research Institute of Biomedical and Health Sciences (IUIBS), University of Las Palmas de Gran Canaria, Las Palmas de Gran Canaria, Spain; 2 Department of Animal Medicine and Surgery, Veterinary Faculty, Complutense University of Madrid, Madrid, Spain

**Keywords:** Heartworm, *Dirofilaria immitis*, Echocardiography, Pulmonary hypertension, Pulmonary artery, Endarteritis

## Abstract

**Background:**

Pulmonary hypertension (PH) is a frequent and severe phenomenon in heartworm disease caused by *Dirofilaria immitis*, mainly caused by intimal proliferation of the arteries and pulmonary thromboembolisms. Transthoracic echocardiography is the method of choice for diagnosing PH in dogs although the diagnosis is often based on indirect and subjective parameters. The Right Pulmonary Artery Distensibility Index (RPAD Index) is a method that has been recently validated to estimate the presence and severity of PH in heartworm-infected dogs. This study compared some echocardiographic parameters commonly used to estimate PH in 93 dogs infected by *D. immitis* and evaluated the impact of the parasite burden, microfilaremia, sex or origin of the dog (client-owned/shelter).

**Results:**

None of the studied echocardiographic variables seemed useful in the estimation of the evaluated clinical aspects, except for the PA/Ao ratio for parasite burden. The RPAD Index was determined in 88 of the dogs; of these, 70.4% had PH (mild: 37.5%, moderate: 19.3%, severe: 13.6%). This Index showed non-significant differences according to microfilaremia, sex, origin or parasite burden. Symptomatic dogs showed PH more often and displayed more severe PH, in addition the presence of symptoms was greater among dogs with high burden; on the other hand 64.4% of asymptomatic dogs had some degree of PH according to the RPAD Index. Apart from the PA/Ao ratio, the other evaluated echocardiographic variables were not useful in evaluating of the hypertensive status of the heartworm-infected dog compared to the RPAD Index.

**Conclusions:**

The estimation of most common indirect parameters is not useful in predicting PH in heartworm-infected dogs. The results confirm the RPAD Index as an objective and supportive test in the monitoring and evaluation of PH in the heartworm-infected dog, and show a potential diagnostic value for the detection of PH in asymptomatic animals.

## Background

Heartworm disease caused by *Dirofilaria immitis* is a cosmopolitan, worldwide distributed vector-borne disease, endemic in areas with high temperatures and humidity which in turn favor the proliferation of the mosquito vector of the infection [1]. *Dirofilaria immitis* mainly affects pulmonary arteries, causing intimal proliferation of the occupied arteries and pulmonary thromboembolisms caused by embolic worm fragments. These events chronically lead to pulmonary hypertension (PH) which is one of the main causes to induce clinical signs and, if not treated, chronically leads to cardiac disease [2].

Transthoracic Doppler echocardiography provides a noninvasive and readily available method for estimating pulmonary arterial pressure in conscious animals and is the method of choice for diagnosing naturally occurring PH in veterinary patients [3]. However, the diagnosis of PH is often based on indirect and subjective parameters, specifically when tricuspid regurgitation and/or pulmonary insufficiency are not present, which only help to partially quantify the disease severity. Recently, the Right Pulmonary Artery Distensibility Index (RPAD Index), which is calculated as the difference in diameter of the right pulmonary artery in systole and diastole as measured by M-mode, was validated as a valuable method for estimating the presence and severity of PH in heartworm-infected dogs [4]. However, this index has not been compared in dogs with *D. immitis* to other indirect measurements commonly used to estimate PH in veterinary patients; nor has its usefulness in evaluating different aspects of the clinical status of infected dogs been established.

On this basis, the aims of this study were (i) to evaluate several common echocardiographic measurements in 2D mode, M-mode and Doppler in dogs infected with *D. immitis*, to determine the sensitivity of the echocardiographic changes in infected dogs in comparison with the RPAD Index, and (ii) to assess the usefulness of the classic echocardiographic measurements and/or RPAD Index in relation to some clinical aspects of the infected dogs. These are presence/absence of symptoms, parasite burden and presence of microfilaremia.

## Methods

In the present study, 93 dogs presented to the Veterinary Teaching Hospital of the University of Las Palmas de Gran Canaria were included. The dogs lived in a hyper-endemic area of *D. immitis* [5, 6]. Of these, 62 were client-owned and 31 were from a local shelter.

None of the animals had received previous treatment for heartworm infection. A complete record was kept for each animal, including identification (age, sex, and breed), clinical history, and demographic data. There were 51 females and 42 males, with a mean age of 5.3 years.

All tested positive to circulating *D. immitis* antigens using a commercial immunochromatographic test kit (Urano test Dirofilaria®, Urano Vet SL, Barcelona, Spain). Dogs were further evaluated for the presence or absence of microfilariae using a modified Knott test.

Dogs underwent echocardiographic examination, using an ultrasound machine with spectral and color Doppler and multifrequency probes (5.5–10 MHz) (Logic P5, General Electric, New York, USA). The dogs were placed in right lateral recumbence with the transducer placed in the third intercostal space. Dogs were conscious and under electrocardiographic monitoring during the whole test. For each measurement, six continuous cardiac cycles were recorded. All the echocardiographic records were carried out by the same researcher.

The presence of worms visible in the pulmonary arteries and right cardiac chambers was assessed. The worm burden was classified according to Venco et al. [7]; a low to high worm burden score ranging from 1 to 4 was assigned as follows: (i) no worms visualized; (ii) few worm echoes in the distal part of the right pulmonary artery; (iii) worm echoes occupying the right pulmonary artery and extending to the main pulmonary artery; and (iv) worm echoes occupying the whole right pulmonary artery and the main pulmonary artery to the level of pulmonary valve.

The following parameters were determined by means of spectral Doppler: pulmonary flow acceleration time (AT), pulmonary flow ejection time (ET), AT/ET ratio, and maximum velocity of the blood flow through the pulmonary artery (PA*Vmax*). Presence or absence of tricuspid regurgitation (TR) and pulmonary regurgitation (PR) as well as the difference gradient between RV/RA and PA/RV were also assessed. By standard two-dimensional mode, the following was determined: main pulmonary artery/aorta ratio (PA/Ao ratio), left atrial (LAV) and right atrial (RAV) volumes, left atrial (LAA) and right atrial (RAA) areas. By M-mode, the measured parameters were: fractional shortening (FS), ejection fraction (EF), right ventricular internal diameter in telediastole (RVIDD) and left ventricular internal diameter in telediastole (LVIDD), RVIDD/LVIDD ratio, right ventricle wall thickness (RVWT), left ventricle posterior wall thickness (LVPWT), RVWT/LVPWT ratio, and tricuspid annular plane systolic excursion (TAPSE). The RPAD Index was determined as described by Venco et al. [4] and was performed in 88 dogs of the present study.

The 88 dogs evaluated by the RPAD Index were further classified into two groups according to presence or absence of symptoms. Dogs were considered as symptomatic when there was presence of one or more symptoms related to heartworm disease (dyspnea, cough, exercise intolerance, weakness, loss of weight, syncope).

The data were analyzed using the SPSS Base 19.0 software for Windows. Continuous variables were expressed as median ± standard deviation according to the Kolmogorov-Smirnov test. Qualitative variables are expressed as percentages. The non-parametric Mann–Whitney *U*-test was used to determine the association between quantitative and categorical variables, along with the Chi square test. In all cases, a *P* value < 0.05 was determined as significant.

## Results

Of the 93 dogs, 52 (55.9%) were microfilaremic and 41 (44.1%) were amicrofilaremic. When parasite burden was evaluated, 75 (80.6%) dogs were considered to have a low parasite burden (score 1 and 2) and 18 (19.4%) were classified as having a high parasite burden (score 3 and 4).

When the presence or absence of microfilariae was compared, there were no statistically significant differences in any of the evaluated echocardiographic measurements. When the dogs were compared by parasite burden, it was observed that only the echocardiographic values of PA/Ao ratio showed statistically significant differences between both groups of dogs (*Z* = -2.004, *P* = 0.045).

Compared by sex, statistically significant differences were found in RVIDD (*Z* = -2.089, *P* = 0.037), LVIDD (*Z* = -2.131, *P* = 0.033), RVIDD/LVIDD (*Z* = -2.563, *P* = 0.01) and LAA (*Z* = -2.803, *P* = 0.005). By origin of the dog (client-owned/shelter), significant differences were found in AT (*Z* = -2.228, *P* = 0.026) and ET (*Z* = -2.965, *P* = 0.003). The mean values of the measured parameters by category are shown in Table 1.

**Table 1:** Echocardiographic parameters measured in the present study

	Microfilaremia		Parasite burden		Sex		Origin		Presence/Absence PH				
	Amicrofilaremic	Microfilaremic	High burden	Low burden	Male	Female	Client-owned	Shelter	RPAD Index ≥ 36%	RPAD Index ≤ 35%	RPAD Index 28–35%	RPAD Index 23–27%	RPAD Index ≤ 22%
*n*	41	52	18	75	42	51	62	31	26	62	33	17	12
AT (ms)	0.08 ± 0.02	0.08 ± 0.02	0.08 ± 0.02	0.08 ± 0.02	0.08 ± 0.02	0.08 ± 0.02	0.08 ± 0.02 ^c^	0.09 ± 0.02 ^c^	0.08 ± 0.02	0.08 ± 0.02	0.07 ± 0.02	0.09 ± 0.02	0.07 ± 0.02
ET (ms)	0.21 ± 0.04	0.22 ± 0.11	0.23 ± 0.05	0.21 ± 0.09	0.22 ± 0.12	0.21 ± 0.05	0.21 ± 0.10 ^c^	0.22 ± 0.03 ^c^	0.23 ± 0.15	0.21 ± 0.05	0.20 ± 0.03	0.22 ± 0.05	0.21 ± 0.08
AT/ET	0.40 ± 0.10	0.37 ± 0.11	0.37 ± 0.11	0.39 ± 0.11	0.40 ± 0.11	0.38 ± 0.11	0.38 ± 0.12	0.39 ± 0.08	0.40 ± 0.09	0.38 ± 0.12	0.37 ± 0.08	0.41 ± 0.14	0.38 ± 0.17
PA*Vmax*	0.80 ± 0.17	0.90 ± 0.31	0.87 ± 0.29	0.82 ± 0.20	0.85 ± 0.20	0.84 ± 0.28	0.85 ± 0.13	0.83 ± 0.22	0.89 ± 0.23	0.83 ± 0.26	0.82 ± 0.19	0.85 ± 0.18	0.84 ± 0.47
PA/Ao	1.02 ± 0.13	1.02 ± 0.15	1.03 ± 0.15^a^	1.01 ± 0.13^a^	1.02 ± 0.17	1.03 ± 0.12	1.01 ± 0.13	1.05 ± 0.17	0.97 ± 0.09 ^d^	1.05 ± 0.15 ^d^	1.03 ± 0.12	1.06 ± 0.17	1.09 ± 0.21
LAV (cm)	7.68 ± 5.79	7.60 ± 5.95	7.85 ± 5.58	7.89 ± 5.90	8.54 ± 5.44	7.17 ± 5.79	7.50 ± 5.56	8.28 ± 5.85	6.98 ± 5.45	8.00 ± 5.68	8.76 ± 6.70	6.30 ± 2.46	8.61 ± 6.13
RAV (cm)	6.16 ± 4.53	5.59 ± 3.64	5.80 ± 3.45	5.86 ± 3.86	6.13 ± 3.60	5.82 ± 4.22	5.83 ± 4.10	6.20 ± 3.68	4.69 ± 3.22	6.43 ± 4.17	6.50 ± 4.07	6.03 ± 3.57	6.91 ± 5.57
LAA (cm^2^)	4.69 ± 2.19	4.33 ± 2.25	4.53 ± 2.10	4.75 ± 2.21	5.20 ± 1.94 ^b^	4.13 ± 2.18 ^b^	4.52 ± 2.17	4.74 ± 2.09	4.44 ± 2.23	4.63 ± 2.09	4.93 ± 2.37	4.42 ± 0.97	4.10 ± 2.58
RAA (cm^2^)	4.33 ± 2.23	4.02 ± 2.28	4.09 ± 2.08	4.20 ± 2.24	4.40 ± 1.70	4.04 ± 2.43	4.27 ± 2.39	4.07 ± 1.62	3.69 ± 1.91	4.42 ± 2.25	4.54 ± 2.52	4.34 ± 1.84	4.22 ± 2.25
FS (%)	39.69 ± 10.64	40.80 ± 7.09	39.85 ± 7.56	39.69 ± 9.26	39.98 ± 9.82	39.61 ± 8.37	39.84 ± 9.13	39.67 ± 8.92	38.13 ± 7.29	40.73 ± 9.71	38.5 ± 7.57	42.73 ± 13.41	44.22 ± 8.27
EF (%)	68.96 ± 12.98	72.37 ± 8.35	71.05 ± 8.86	69.77 ± 11.22	69.64 ± 11.49	70.53 ± 10.43	70.11 ± 10.85	70.14 ± 11.12	69.06 ± 9.11	70.83 ± 11.70	68.84 ± 9.38	70.98 ± 16.52	76.09 ± 8.74
RVIDD (cm)	1.03 ± 0.53	0.92 ± 0.42	0.93 ± 0.43	0.96 ± 0.46	0.85 ± 0.39 ^b^	1.07 ± 0.51 ^b^	0.94 ± 0.49	1.04 ± 0.45	0.91 ± 0.42	1.02 ± 0.50	0.98 ± 0.49	1.09 ± 0.40	1.06 ± 0.65
LVIDD (cm)	2.84 ± 0.87	2.99 ± 0.80	3.00 ± 0.75	3.00 ± 0.79	3.14 ± 0.82 ^b^	2.76 ± 0.76 ^b^	2.91 ± 0.82	2.98 ± 0.8	2.79 ± 0.80	2.99 ± 0.81	3.00 ± 0.86	2.96 ± 0.65	3.01 ± 0.92
RVIDD/LVIDD	0.40 ± 0.30	0.33 ± 0.18	0.33 ± 0.17	0.34 ± 0.17	0.30 ± 0.17 ^b^	0.42 ± 0.27 ^b^	0.36 ± 0.27	0.37 ± 0.17	0.35 ± 0.18	0.37 ± 0.27	0.35 ± 0.19	0.38 ± 0.15	0.44 ± 0.50
RVWT (cm)	0.60 ± 0.15	0.57 ± 0.20	0.55 ± 0.19	0.56 ± 0.17	0.56 ± 0.16	0.59 ± 0.18	0.57 ± 0.18	0.59 ± 0.16	0.56 ± 0.15	0.58 ± 0.18	0.57 ± 0.18	0.61 ± 0.16	0.56 ± 0.23
LVPWT (cm)	0.87 ± 0.23	0.85 ± 0.23	0.85 ± 0.24	0.87 ± 0.24	0.86 ± 0.25	0.86 ± 0.22	0.88 ± 0.26	0.82 ± 0.18	0.82 ± 0.23	0.88 ± 0.24	0.89 ± 0.22	0.82 ± 0.21	0.92 ± 0.33
RVWT/LVPWT	0.72 ± 0.20	0.68 ± 0.21	0.66 ± 0.20	0.67 ± 0.21	0.68 ± 0.21	0.69 ± 0.20	0.67 ± 0.22	0.72 ± 0.16	0.72 ± 0.21	0.68 ± 0.20	0.66 ± 0.19	0.76 ± 0.19	0.66 ± 0.22
TAPSE (cm)	1.34 ± 0.38	1.39 ± 0.32	1.36 ± 0.30	1.35 ± 0.33	1.35 ± 0.34	1.36 ± 0.33	1.38 ± 0.36	1.29 ± 0.26	1.43 ± 0.36	1.32 ± 0.32	1.34 ± 0.36	1.35 ± 0.24	1.23 ± 0.27
RPAD Index (%)	32.77 ± 8.67	32.42 ± 10.22	32.96 ± 12.2	32.73 ± 8.85	32.67 ± 9.50	32.51 ± 9.62	32.48 ± 9.54	32.77 ± 9.64	43.58 ± 6.58^d^	27.97 ± 6.19^d^	32.71 ± 2.19^d^	25.87 ± 1.42^d^	17.89 ± 3.21^d^

Statistically significant differences (*P* < 0.05) compared by parasite burden (^a^), sex (^b^), origin (^c^) and presence/absence of pulmonary hypertension (^d^)

*Abbreviations*: *AT* pulmonary artery flow acceleration time, *ET* pulmonary artery flow ejection time, *PAVmax* maximum velocity of the blood flow through pulmonary artery, *PA/Ao* main pulmonary artery/aorta ratio, *LAV* left atrial volume, *RAV* right atrial volume, *LAA* left atrial area, *RAA* right atrial area, *FS* fractional shortening, *EF* ejection fraction, *RVIDD* right ventricular internal diameter in telediastole, *LVIDD* left ventricular internal diameter in telediastole, *RVWT* right ventricular wall thickness, *LVPWT* left ventricular posterior wall thickness, *TAPSE* tricuspid annular plane systolic excursion, *RPAD Index* Right Pulmonary Artery Distensibility Index, *PH* pulmonary hypertension

Absence or presence of PH, as well as the severity, was based on the determination of the RPAD Index. According to Venco et al. [4], a RPAD Index between 35% and 28% was correlated with mild PH (30–55 mm Hg), between 27% and 23% with moderate PH (56–79 mm Hg) and between 22% or less with severe PH (> 79 mm Hg). Of the dogs measured (*n* = 88), 29.6% (26/88) did not have PH, and 70.4% (62/88) were hypertensive, showing mild PH 37.5% (33/88), moderate PH 19.3% (17/88) and severe PH 13.6% (12/88).

When the dogs were compared by PH status (normotensive *vs* hypertensive), none of the echocardiographic parameters showed statistically significant differences between groups, except the PA/Ao ratio (*Z* = -2.310, *P* = 0.02). On the other hand, when the dogs where compared by severity of PH, no statistically significant differences were found in any of the studied parameters. The TAPSE value was abnormal in all animals, regardless of the presence or absence of PH.

TR and/or PR were present in 27 dogs. The gradients were not consistent with pulmonary hypertension whether this be systolic or diastolic. PR was present in 25 dogs (mean velocity flow: 1.24 ± 0.87 m/s; mean gradient: 9.16 ± 12.76 mm Hg) and TR was present in 11 dogs (mean velocity flow: 1.35 ± 0.93 m/s; mean gradient: 11.25 ± 14.88 mm Hg). The mean RPAD Index for dogs with TR was 29.9 ± 7.8%, and for dogs with PR was 27.9 ± 7.9%.

The RPAD Index showed not statistically significant differences according to parasite burden, microfilaremic status, sex or origin of the animals. When presence/absence of symptoms was evaluated, a statistically significant difference was observed in the RPAD Index between symptomatic dogs (*n* = 29; mean RPAD Index: 26.8 ± 9.3%) and asymptomatic dogs (*n* = 59; mean RPAD Index: 35.4 ± 8.3%) (*Z* = -3942, *P* < 0.0001) (Table 2). Furthermore, a significant difference was found between presence/absence of symptoms and the parasite burden (*χ* ^2^ = 8.394, *df* = 1, *P* = 0.005) (Table 3).

**Table 2:** Dogs classified by the RPAD Index and according to the presence/absence of symptoms and by parasite burden. Data are shown as number of dogs (*n*). Animals were considered having mild pulmonary hypertension (30–55 mm Hg) with a RPAD Index between 28% and 35%, moderate pulmonary hypertension (56–79 mm Hg) with a RPAD Index between 23% and 27% and severe pulmonary hypertension (> 79 mm Hg) with a RPAD Index ≤ 22%. When the RPAD Index was ≥36% the dog was considered normotensive

	Symptomatic	Asymptomatic	High burden	Low burden
Normotensive (*n* = 26)	5	21	8	18
Hypertensive (*n* = 62)	24	38	17	45
Mild PH (*n* = 33)	5	28	8	25
Moderate PH (*n* = 17)	8	9	4	13
Severe PH (*n* = 12)	11	1	5	7
TOTAL (*n* = 88)	29	59	25	63

*Abbreviation*: *PH* pulmonary hypertension

**Table 3:** Dogs compared by presence/absence of symptoms and by parasite burden (high burden/low burden). Data are shown as number of dogs (*n*)

	Symptomatic	Asymptomatic
High burden (*n* = 25)	14	11
Low burden (*n* = 63)	15	48
Total (*n* = 88)	29	59

## Discussion

In heartworm disease, embolism of dead heartworms can cause acute clinical signs of PH, while proliferative intimal changes lead to irreversible structural damage to the vasculature and sustained PH [2, 8]. An objective determination of PH is important given that it is a frequent and severe phenomenon in heartworm disease.

Except for the PA/Ao ratio, the results showed no significant differences in the studied echocardiographic parameters between dogs with PH and without PH measured by RPAD Index; moreover, there were no significant differences in any parameter by degrees of PH. Traditionally, the evaluation of the presence of PH in dogs has been determined from clinical and indirect echocardiographic findings when tricuspid regurgitation and/or pulmonary insufficiency were not present [3]. Previous reports have studied the diagnostic value of AT, AT: ET, and PA/Ao ratio in predicting PH [9–11]; although some accuracy was found, the authors indicated that the described variables were hindered by relatively low sensitivity and specificity values (PA/Ao ratio) or technical and alignment limitations (AT, AT/ET ratio); studies also concluded that the RPAD Index may be useful and predictive of PH in dogs when tricuspid regurgitation is absent [11]. These observations are in accordance with the results from the present study, which showed that only the PA/Ao ratio presented some significance when comparing normotensive dogs versus hypertensive dogs, but this significance disappeared when hypertensive dogs were evaluated by severity of PH. Furthermore, based on the results, the estimation of most common indirect parameters was not useful in predicting PH in heartworm-infected dogs against the RPAD Index and showed that the estimation of the studied echocardiographic measurements presented little usefulness when comparing parasite burden, presence of microfilaremia, sex or origin of the animal.

The PA/Ao ratio was also significantly greater in dogs with high parasite burden in comparison with dogs with low burden; however, the parasite burden seems to have no effect in the RPAD Index. This difference could be due to the mechanical pressure caused by the presence of a larger amount of parasites in the pulmonary arteries. However further studies should be carried out to evaluate this conclusion.

Significant differences were found in the AT and ET parameters as regards the origin of the animal (client owned/shelter). There is not a clear explanation for this; some authors reported that, in addition to the pulmonary pressure, the ET and AT can be influenced by other factors like heart rate, cardiac output or function of the right ventricle [12]. Also, the ET can be influenced because the accuracy of the flow velocities can be affected by its anatomical displacement in patients with dilated pulmonary arteries and high pulsed-wave Doppler gain which might underestimate exact time measurements [13]. Furthermore, Visser et al. [11] described that correct measurements of AT and ET can be difficult to acquire in dyspneic dogs.

The differences found in the results of RVIDD, LVIDD, RVIDD/LVIDD and LAA by sex could be attributed to physiological differences in the size of the animals.

All studied dogs showed an abnormal TAPSE value, an echocardiographic measurement that allows the assessment of the right ventricular systolic function which is a strong predictor of outcome in human patients with PH [14]. It has also been described that patients with either left or right ventricular systolic dysfunction had statistically smaller TAPSE than patients with normal biventricular systolic function [15].

It was observed that the dogs showing any symptom compatible with heartworm disease showed PH more often and in general more severely than the asymptomatic dogs. Similarly, the presence of symptoms was more prevalent among dogs with high parasite burden. It seems reasonable to associate the presence of some symptoms with the consequences of high pulmonary pressure; also, a high parasite burden has been linked to high levels of serum biomarkers of cardiopulmonary and inflammatory damage in dogs with *D. immitis* [16–18]. More importantly, just as not all the symptomatic dogs from the present study were hypertensive, the results showed that a high percentage of asymptomatic dogs had some degree of PH. This highlights the importance of early detection of this condition by objective methods, which could allow appropriate therapeutic measures aimed at minimizing the evolution of the vascular damage.

Moreover, the RPAD Index can be seen as a more sensitive marker for PH since, although 27 of the 62 hypertensive dogs showed presence TR and/or PR, none of them showed values consistent with pulmonary hypertension.

No significant changes were observed in the RPAD Index regarding the microfilaremic status, sex or origin of the dog. In addition, the parasite burden did not significantly affect the RPAD Index. Furthermore, the results showed a high percentage of dogs with PH and low worm burden, which is in accordance with previous observations made by other authors who reported that the severity of hypertension does not correlate with the number of parasitic worms and factors such as intensity of exercise and lower parasite burden in older dogs, this being due to natural death of the worms [7, 19]. Other factors that may contribute to the vascular endarteritis and therefore development of hypertension are currently being studied [20].

## Conclusions

Based on these results, none of the classic echocardiographic parameters studied seemed useful in estimating the severity of PH and other clinical aspects. Moreover, these echocardiographic variables did not seem to offer any additional benefits in the evaluation of the hypertensive status of the heartworm-infected dog compared to the RPAD Index, which previously showed a strong correlation with invasive pulmonary arterial pressures demonstrating its potential diagnostic value in dogs [4]. The results confirm the RPAD Index as an objective and supportive test in the monitoring and evaluation of the heartworm-infected dog and show its diagnostic value in the early detection of PH in asymptomatic animals, which could facilitate appropriate therapeutic measures aimed at minimizing the evolution of the vascular damage. Although additional studies would be necessary to consolidate these conclusions, these results encourage the use of the RPAD Index as a standardized method for assessing the hypertensive status of the heartworm-infected dogs.

## **Abbreviations**

AT: Pulmonary flow acceleration time
EF: Ejection fraction
ET: Pulmonary flow ejection time
FS: Fractional shortening
LAA: Left atrial area
LAV: Left atrial volume
LVIDD: Left ventricular internal diameter in telediastole
LVPWT: Left ventricle posterior wall thickness
PA/Ao ratio: Main pulmonary artery/aorta ratio
PAV max: Maximum velocity of the blood flow through the pulmonary artery
PH: Pulmonary hypertension
PR: Pulmonary regurgitation
RA: Right atrium
RAA: Right atrial area
RAV: Right atrial volume
RPAD Index: Right Pulmonary Artery Distensibility Index
RV: Right ventricle
RVIDD: Right ventricular internal diameter in telediastole
RVWT: Right ventricle wall thickness
TAPSE: Tricuspid annular plane systolic excursion
TR: Tricuspid regurgitation
